# Rapid Synthesis and Formation Mechanism of Core-Shell-Structured La-Doped SrTiO_3_ with a Nb-Doped Shell

**DOI:** 10.3390/ma8073992

**Published:** 2015-07-02

**Authors:** Nam-Hee Park, Takafumi Akamatsu, Toshio Itoh, Noriya Izu, Woosuck Shin

**Affiliations:** National Institute of Advanced Industrial Science and Technology (AIST), Shimo-Shidami, Moriyama-ku, Nagoya 463-8560, Japan; E-Mails: zznami215@gmail.com (N.-H.P.); t-akamatsu@aist.go.jp (T.A.); itoh-toshio@aist.go.jp (T.I.); n-izu@aist.go.jp (N.I.)

**Keywords:** La-doped SrTiO_3_, nanocubes, Nb surface doping, hydrothermal method, core-shell structure, thermoelectric materials

## Abstract

To provide a convenient and practical synthesis process for metal ion doping on the surface of nanoparticles in an assembled nanostructure, core-shell-structured La-doped SrTiO_3_ nanocubes with a Nb-doped surface layer were synthesized via a rapid synthesis combining a rapid sol-precipitation and hydrothermal process. The La-doped SrTiO_3_ nanocubes were formed at room temperature by a rapid dissolution of NaOH pellets during the rapid sol-precipitation process, and the Nb-doped surface (shell) along with Nb-rich edges formed on the core nanocubes via the hydrothermal process. The formation mechanism of the core-shell-structured nanocubes and their shape evolution as a function of the Nb doping level were investigated. The synthesized core-shell-structured nanocubes could be arranged face-to-face on a SiO_2_/Si substrate by a slow evaporation process, and this nanostructured 10 μm thick thin film showed a smooth surface.

## 1. Introduction

Perovskite-type oxides are an incredibly fascinating material due to their widespread applications in electronics, energy storage devices, catalysts, and sensors [1,2,3,4,5,6]. SrTiO_3_ is a typical perovskite-structured oxide with a large bandgap of 3.2 eV [7,8,9], which can have its chemical and electrical properties tuned either by doping or by controlling the number of oxygen vacancies [10,11]. It is well known that the properties of SrTiO_3_ nanoparticles strongly depend on their morphology, structure, chemical composition, and crystallinity [12,13]. The development of new synthesis techniques and structures for SrTiO_3_ materials is essential in view of their practical applications. In particular, ordered nanoparticle superstructures and three-dimensional (3D) architectures have attracted extensive attention because of their large specific areas, number of interface and active sites for transport, and reactions such as mesocrystals and supernanoparticles [14,15,16,17]. For these reasons, a cubic morphology has attracted extensive interest, as it is considered the ideal candidate for forming close-packed ordered structures and 3D architectures.

Recently, we proposed a 3D bulk material model consisting of nanoscale La-doped SrTiO_3_ nanocube grains and Nb-doped grain boundaries for n-type thermoelectric materials formed using a nanostructuring approach, with a predicted high thermoelectric performance [18]. In our previous work on realizing the proposed 3D model structure via solution synthesis, we synthesized SrTiO_3_ mesocrystals with a well-defined cubic shape 60 nm in size through an oriented attachment and crystallographic fusion of small scale primary SrTiO_3_ nanoparticles using an organic material [19], and the synthesis process of La-doped SrTiO_3_ nanocubes having a core-shell structure have been suggested for doping its surface using the metal ions [20]. A particulate-based thin film of self-assembled 5% La-doped SrTiO_3_ nanocubes with a size of *ca.* 15 nm have also been fabricated on a Si substrate using an organic material instead of a bulk material, with their thermoelectric performance measured [21]. Despite the success and preparation efforts in SrTiO_3_-based 3D bulk structures or thin films, it is still necessary to develop a process for Nb doping at the grain boundaries of SrTiO_3_-based 3D bulk structures for use in practical applications. The formation of Nb-doped grain boundaries are important because these grain boundaries form a two-dimensional electron gas (2DEG)-causing energy filtering effect, which will enhance the power factor of the nanocube grains, effectively lowering the lattice thermal conductivity [19,22].

In this study, we demonstrate a Nb-doping process on the surface of La-doped SrTiO_3_ nanocubes with a core-shell structure. The La-doped SrTiO_3_ nanocubes with Nb surface doping are synthesized via a rapid synthesis process combining a rapid sol-precipitation and hydrothermal method to change the doping levels of Nb and La on the shell and core, respectively. The formation mechanism and evolution of the core-shell-structured nanocube morphology as a function of Nb doping level are also investigated. Furthermore, to obtain a spontaneously assembled nanocube structure for thermoelectric applications, thin films are fabricated by a slow evaporation process on a SiO_2_/Si substrate.

## 2. Results and Discussion

The abbreviations for the synthesized Nb-LaSTO nanocubes with various doping levels of Nb and La (0%–20%) are listed in Table 1.

**Table 1:** Abbreviations for the synthesized Nb-LaSTO nanocubes with varied doping levels of Nb and La (0%–20%).

Nb and La doping levels		Nominal La doping levels (mol%)		
		0	10	20
Nominal Nb doping levels (mol%)	0	STO	10STO	20STO
	5	5Nb-STO	5Nb-10STO	5Nb-20STO
	10	10Nb-STO	10Nb-10STO	10Nb-20STO
	20	20Nb-STO	20Nb-20STO	20Nb-20STO

**Figure 1:**
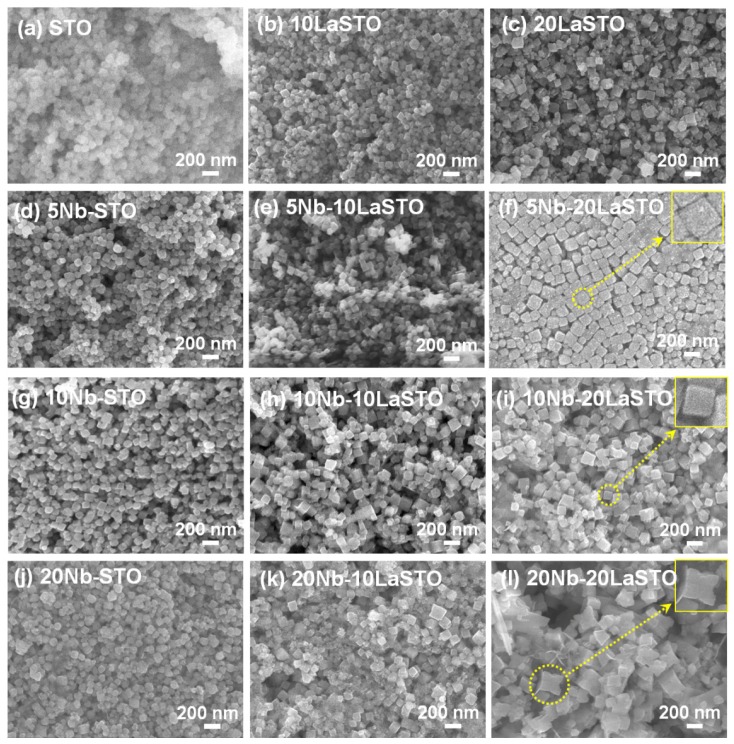
FE-SEM images of the STO and core-shell-structured Nb-LaSTO nanocubes with various Nb and La doping levels. Scale bar = 200 nm.

Figure 1 shows the FE-SEM images of the Nb-LaSTO nanocubes deposited on SiO_2_/Si substrates with varied Nb and La doping levels. When the doping levels of the Nb and La were 0% (STO), the STO nanoparticles were spherical in shape and approximately 100 nm in size, as shown in Figure 1a. The spherical shaped STO nanoparticles were changed to a cubic structure, with the size increasing as the La doping level in the LaSTO, as shown in Figure 1b,c.

Similarly, the Nb-doped STO (Nb-STO) nanoparticles formed cubic shapes enclosed entirely by (100) facets with increasing Nb doping levels, as shown in Figure 1d,g,j. The La and Nb doping facilitated the formation of a cubic morphology. When 5% Nb was doped on the surface of the cubic-shaped 10LaSTO and 20LaSTO cores (labelled as the 5Nb-10LaSTO and 5Nb-20LaSTO nanoparticles) show not only uniform cubic shapes and sizes, but also sharp edges, as shown in Figure 1e,f; furthermore, the 5Nb-20LaSTO nanocubes show a face-to-face arrangement, shown in Figure 1f. As the Nb doping level increased to over 10% on the surface of the 10LaSTO and 20LaSTO samples (Figure 1h,i,k,l), the nanocubes lost their uniformity in shape and size. The 20Nb-10LaSTO and 20Nb-20LaSTO nanoparticles have a concave cubic shape, large in size, as shown in Figure 1k,l, indicating a fast growth along the (111) direction of the nanocubes under a hydrothermal process. This result indicates that the newly formed Nb-doped surfaces (*i.e.*, the shell) grew preferentially on the (111) plane of the LaSTO core nanocubes as low surface energy (100) facets under the hydrothermal growth condition, leading to the formation of cubic shapes with sharp edges when the Nb doping level was below 10%, and concave cubic shapes at a high Nb doping level of 20%.

**Figure 2:**
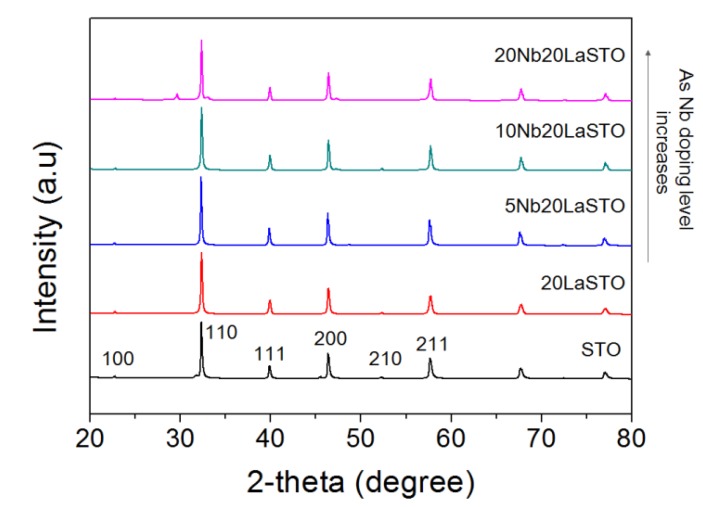
XRD patterns of core-shell-structured 20LaSTO nanocubes with various Nb doping levels.

Figure 2 shows the XRD patterns of a series of 20LaSTO nanocubes with different Nb doping levels. All peaks in the XRD patterns correspond to those of a perovskite-type SrTiO_3_ structure. Minor peaks between 28 to 48 degrees in XRD of Figure 2 are corresponded to NaCl. NaOH pellet and TiCl_4_ as starting materials forms NaCl. It may be not enough to wash for removing NaCl in the specimens. No evidence was found for the presence of impurities in any of the nanoparticles.

**Figure 3:**
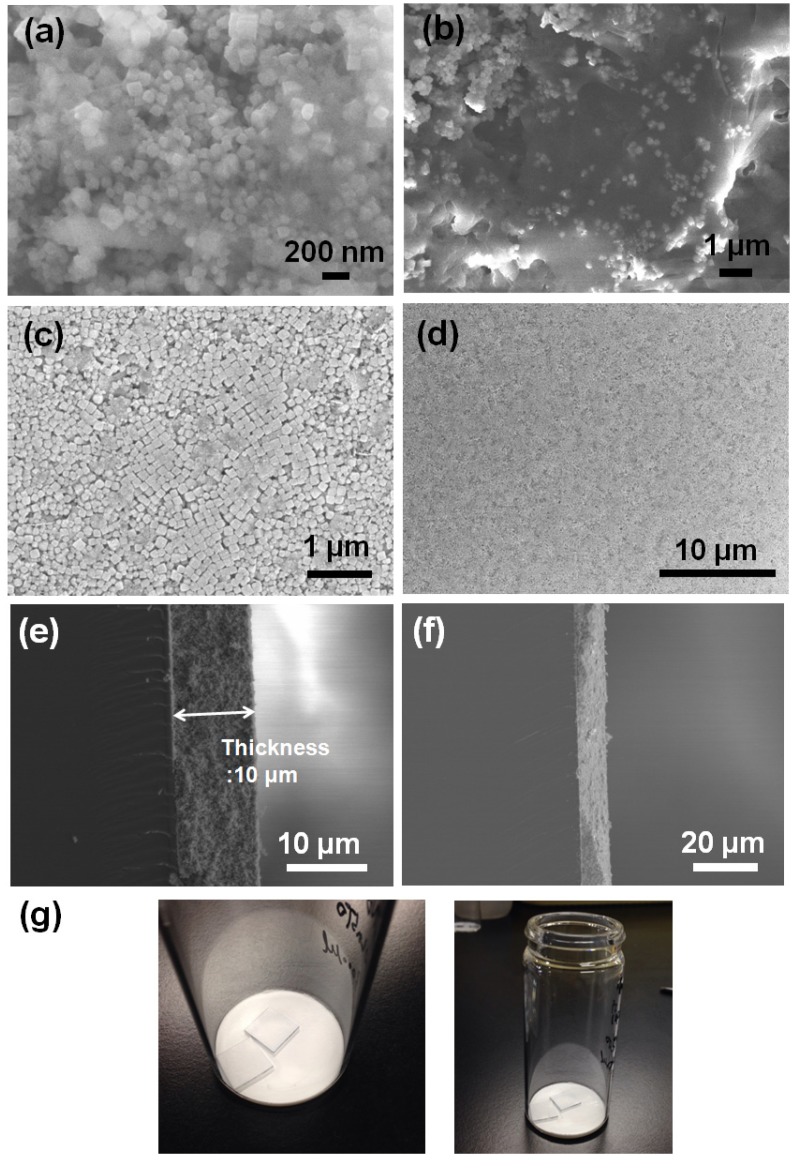
(**a**–**f**) FE-SEM images at different magnifications. 5Nb-20LaSTO nanocubes obtained (**a**,**b**) after the sol-precipitation process of Stage 1 and (**c**,**d**) after the hydrothermal treatment of Stage 2; (**e**,**f**) Cross-section view of the thin film of self-assembled 5Nb20LaSTO nanocubes deposited on a SiO_2_/Si substrate by the slow evaporation process; (**g**) Photographs of the self-assembled thin films comprising the core-shell-structured 5Nb20LaSTO nanocubes prepared by the slow evaporation process.

**Figure 4:**
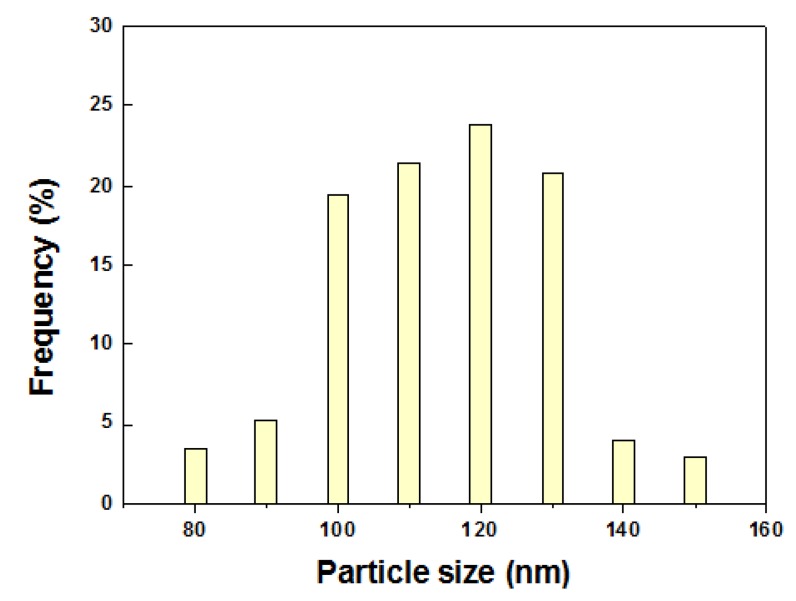
The size distribution graph of the 5Nb-20LaSTO nanocubesin the FE-SEM image of Figure 3c. The size of the nanocubes was estimated using 155 nanocubes.

Figure 3 shows the FE-SEM images at different magnifications of 5Nb-20LaSTO nanocubes obtained before and after the hydrothermal growth, in addition to cross-sectional FE-SEM images of thin films fabricated from these nanocubes by a slow evaporation process. Cubic-shaped nanoparticles with the Nb-containing precursor covering them were observed, as shown in Figure 3a,b, after the rapid sol-precipitation process of Stage 1 at room temperature and addition of the Nb precursor, although the formation of the SrTiO_3_ phase requires a high pH and high temperature in aqueous solution. The LaSTO nanocubes formed rapidly due to the violent exothermic reaction of the NaOH pellet dissolution, producing a high pH and high temperature. After the hydrothermal process of Stage 2 (Figure 3c,d), only cubic-shaped nanoparticles were observed, with the unreacted precursor that surrounded the nanocubes having vanished compared to nanocubes collected prior to the hydrothermal process. Furthermore, the 5Nb-20LaSTO nanocubes showed a face-to-face assembly, forming intimate contacts with one another on the SiO_2_/Si substrate, of *ca.* 134.5 nm in size, which was reliably estimated from the FE-SEM image. The size distribution graph of the 5Nb-20LaSTO nanocubes in Figure 3c is shown in Figure 4. The low-magnification FE-SEM image in Figure 3d shows a large area of the assembled nanocubes with no cracks observed. The thickness of the thin film is 10 μm (Figure 3e), showing a smooth and flat surface (Figure 3f). Photographs of thin films comprised of the 5Nb-20LaSTO nanocubes, fabricated by a slow evaporation process, are shown in Figure 3g.

**Figure 5:**
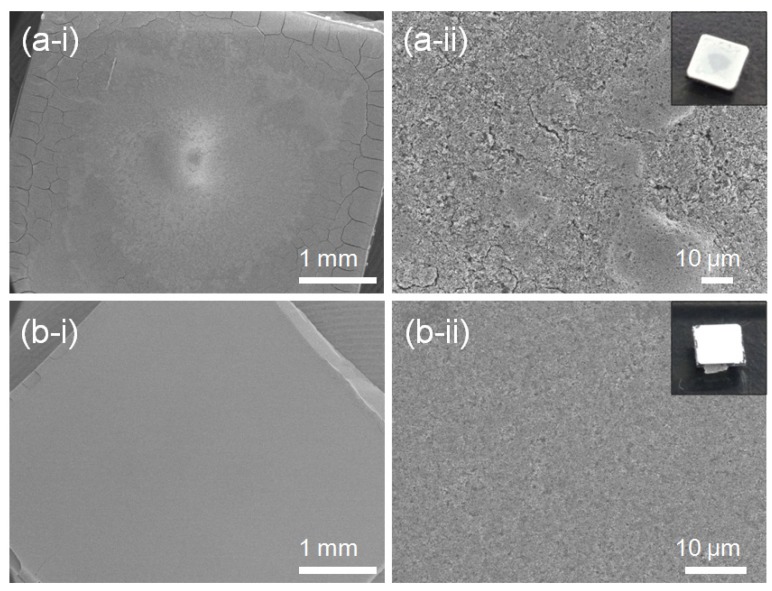
FE-SEM images and photographs of 20Nb-STO nanoparticle thin films prepared by an evaporation process (**a**) in vacuum at 50 °C and (**b**) in air at room temperature.

Evaporation-rate dependent cracks and a rough surface are observed in FE-SEM images and photographs of the 5Nb-STO (Figure 5). The thin film in Figure 5a was fabricated by evaporating the solution over a few minutes in a vacuum oven at 50 °C. When the dispersed solution of nanocubes evaporated quickly over a few minutes under vacuum, a cracked thin film and rough surface were observed compared to the thin film evaporated in air at room temperature (Figure 5b).

**Figure 6:**
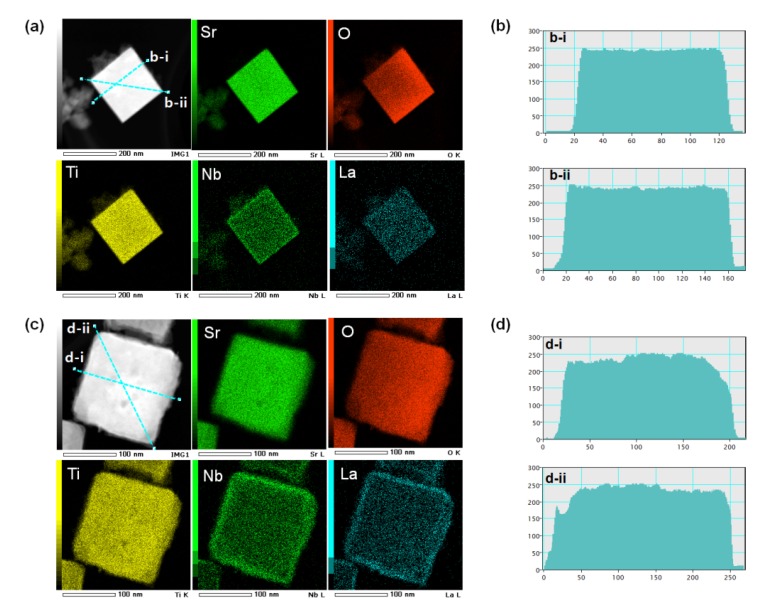
STEM images, elemental maps, and image intensity profiles of core-shell-structured 5Nb-20LaSTO nanocubes obtained (**a**,**b**) before the hydrothermal process and (**c**,**d**) after the hydrothermal process. The different colors in the elemental map indicate the elemental distribution of Sr, O, Ti, Nb, and La in the bright-field STEM images. The image intensity profile measured across an obtained nanocube in the dark-field STEM images have the selected regions marked by a blue line.

High-angle annular dark-field scanning transmission electron microscopy (HAADF-STEM) images, elemental mapping, and image intensity profiles of 5Nb-20LaSTO nanocubes after the rapid sol-precipitation and after the hydrothermal step are shown in Figure 6. The image intensity profile, measured across an obtained nanocube in the dark-field STEM image, has the measured regions noted by a blue line. Figure 6a shows the bright-field STEM image and elemental mapping results of a 5Nb-20LaSTO nanocube collected after the rapid sol-precipitation process (Stage 1) with the addition of the Nb precursor. The nanocube shows sharp edges, and unreacted precursor solution near the nanocube was observed, as shown by the arrows, corresponding to the FE-SEM images of Figure 2a,b. According to the elemental mapping, Sr, O, Ti, Nb, and La were distributed throughout the entire nanocube after the rapid sol-precipitation. However, the Nb can be assumed to cover the nanocube surface, since the Nb was added and dissolved after the rapid sol-precipitation process of Stage 1 finished. The image intensity profile from the dark-field STEM image of the nanocube in Figure 5a revealed it was around 100 nm in size with a smooth surface and sharp edges, as shown in Figure 5b-i,b-ii.

After the hydrothermal process, the core-shell structure was formed, and the precursor solution was no longer observed. The thickness of the Nb-dopant surface layer (shell) was approximately 12 nm, as shown in the bright-field STEM image of Figure 6c. La, Sr, Ti, O, and Nb are all present in the corresponding elemental mapping. The elemental mapping reveals that the Nb was concentrated at the edges of the nanocube, forming Nb-rich edges. Based on the intensity profile from the dark-field STEM image of the nanocube in Figure 6c, slightly rough surfaces and a larger size (around 180 nm) are evident, as shown in Figure 6d, indicating that the dissolved Nb ions from the precursor solution are transformed into a surface layer (shell) on the LaSTO nanocube, while maintaining the cubic shape. These results provide evidence for the Nb doping the surface and the Nb-rich edges of a nanocube in particular after the hydrothermal process.

**Figure 7:**
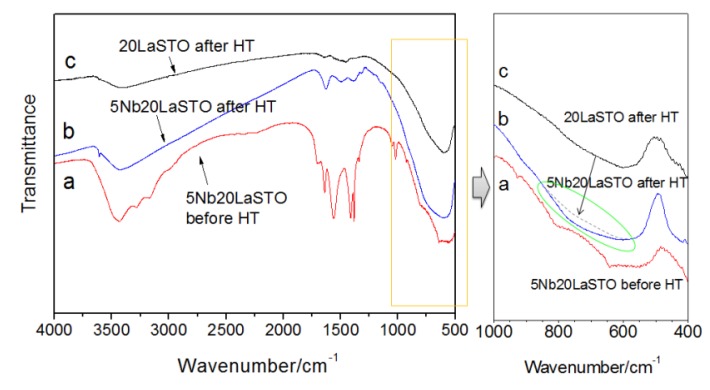
FT-IR spectra of the core-shell-structured 5Nb20LaSTO nanocubes obtained (**a**) after the rapid sol-precipitation method (Stage 1); (**b**) after the hydrothermal treatment (Stage 2); and (**c**) of 20LaSTO nanocubes obtained after the hydrothermal treatment.

Figure 7 shows the FT-IR spectra of core-shell-structured 5Nb-20LaSTO nanocubes before and after the hydrothermal process, as well as 20LaSTO nanocubes obtained after the hydrothermal method. The FT-IR spectra analysis ranged from 4000–400 cm^−1^. The absorption band at 3430 cm^−1^, of the OH group, was observed in the spectra of all three samples. As shown in Figure 7a, absorption bands ascribed to the N–H, C=O, C–C, and C–O bands from the Nb precursor appeared in the range of 3300–3100 cm^−1^ and 1680–1380 cm^−1^. However, most of the bands reduced or disappeared after the hydrothermal process (Figure 7b), likely due to changed interactions between the 20LaSTO surface and the adsorbed Nb starting material as the hydrothermal step progressed. The FT-IR analysis result in the range of 1000–400 cm^−1^ further confirms this, as besides the TiO_6_ octahedron stretching mode at approximately 600 cm^−1^ in the 20LaSTO (shown in Figure 7c), an additional peak derived from the difference between the 20LaSTO and 5Nb20LaSTO spectra obtained after the hydrothermal process (Figure 7b), was observed in the range of 600 to 950 cm^−1^, which can be assigned to the Nb–O vibration mode due to the substitution of the Ti sites with Nb [23,24].

**Figure 8:**
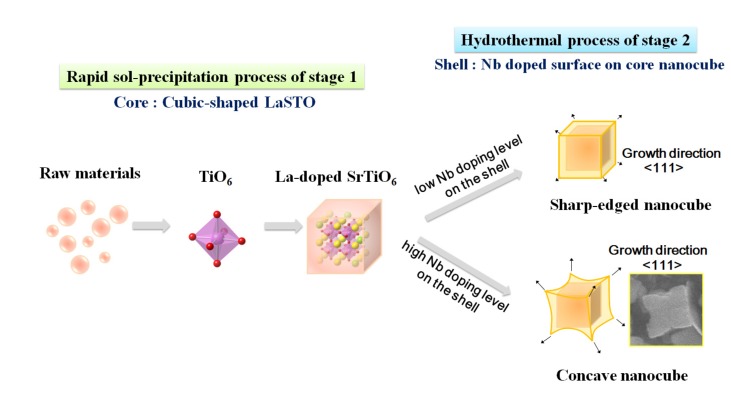
Schematic of the core-shell-structured LaSTO nanocube formation mechanism with Nb doping of the surface, and their shape evolution as a function of Nb doping level.

Figure 8 is a schematic showing the formation mechanism of the core-shell-structured Nb-LaSTO nanocubes produced by a rapid process via a combined rapid sol-precipitation and hydrothermal process, along with the shape evolution as a function of the Nb doping levels. Initially, the LaSTO nanocubes (cores) rapidly formed at room temperature upon the dissolution of NaOH pellets via a rapid sol-precipitation process, confirmed by FE-SEM images (Figure 3a,b), STEM images, elemental mapping, and image intensity profiles (Figure 6a,b). Subsequently, the unreacted precursor solution of the Nb source transforms into the surface layer (shell) on the core nanocubes under the hydrothermal process, as confirmed by the STEM image, elemental mapping, and image intensity profile (Figure 6c,d). The shape evolution was also investigated relative to increased Nb doping levels, as shown in the FE-SEM images of Figure 2. With Nb doping levels below 10%, the LaSTO nanocubes grew preferentially along the (111) direction and formed sharp edges. At the same time, a higher Nb doping level of 20% resulted in a faster growth rate on the (111) planes of the nanocubes, finally forming concave nanocubes.

## 3. Experimental Section

**Figure 9:**
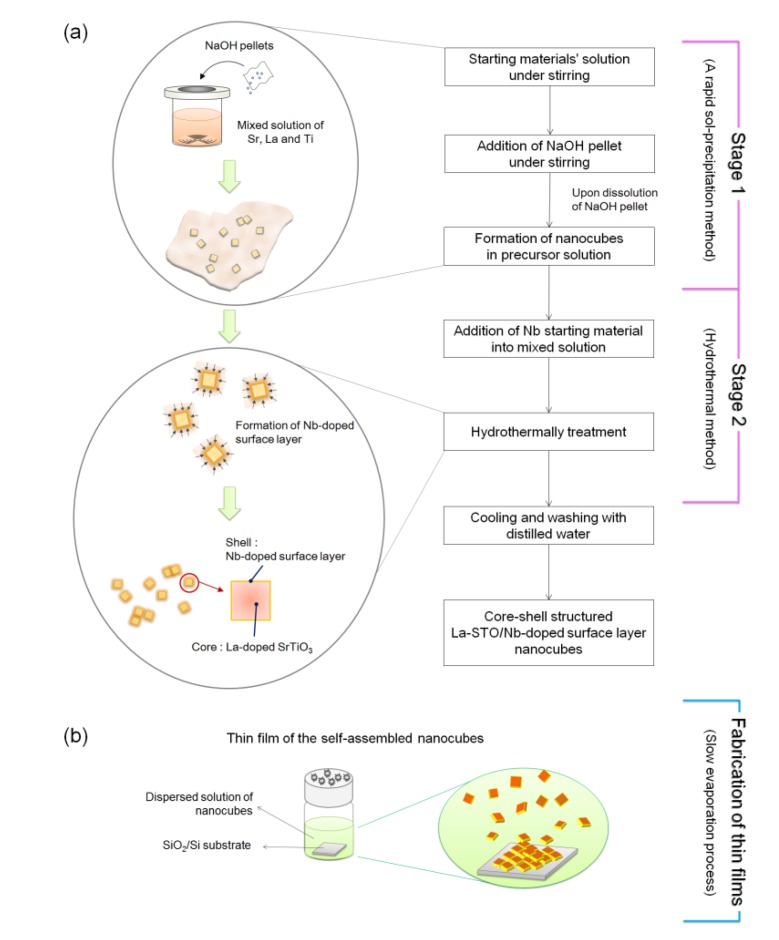
Schematic of the formation mechanism and experimental procedure of (**a**) core-shell-structured Nb-LaSTO nanocubes synthesized via a rapid synthesis combining a rapid sol-precipitation and hydrothermal method; and (**b**) the self-assembled thin films fabricated by a slow evaporation process.

Figure 9 has a schematic illustrating the formation mechanism and experimental procedure used to form the core-shell-structured La-doped SrTiO_3_ nanocubes with the Nb-doped surface layer, synthesized by a rapid process combining a rapid sol-precipitation method (Stage1) and hydrothermal method (Stage 2).

In Stage 1, the La-doped SrTiO_3_ nanocubes (LaSTO) were obtained at room temperature by a rapid sol-precipitation method. Both Sr(OH)_2_·8H_2_O and La(NO_3_)_2_·6H_2_O (0%–20%) were dissolved in a 40 mL mixed solution of ethanol and distilled water containing 2.52 mL of acetic acid. TiCl_4_ (0.13 M,(La+Sr):(Ti+Nb) = 1:1) was added to 16 mL of ethanol under mechanical stirring. The two prepared solutions were then mixed under stirring. Then, 3.5 g of sodium hydroxide pellets was added to the mixture as a precipitating agent and to adjust the pH (~14). Upon dissolution of the sodium hydroxide pellets, the viscosity of the solution continuously increased with the color rapidly changing to white, indicating the formation of a precipitate as a white suspension. In Stage 2, ammonium niobate oxalate hydrate (5%–20%) was subsequently added into the solution as the water-soluble Nb precursor to dope the Nb onto the surface of the LaSTO nanocube shells, with the solution then transferred to a Teflon-lined autoclave. The sealed autoclave was heated to 200 °C and kept for 24 h under mechanical stirring to form the Nb-doped surface on the La-doped SrTiO_3_ nanocubes (Nb-LaSTO), followed by cooling to room temperature. The synthesized nanocubes were collected by centrifugation and washed twice with distilled water. For preparation of thin films, the obtained nanocubes were dispersed in distilled water and deposited on a 1.0 × 1.0 cm^2^ SiO_2_/Si substrate with the water allowed to evaporate slowly in air for a week.

The obtained Nb-LaSTO nanocubes and thin films were characterized by an X-ray diffractometer (XRD, Rigaku, Smartlab), field emission scanning electron microscopy (FE-SEM, JEOL, JSM-6335FM), scanningtransmission electron microscopy (STEM, JEOL, JEM-2100F), and Fourier transform infrared spectroscopy (FT-IR, JASCO, FT/IR-610).

## 4. Conclusions

A rapid process combining a rapid sol-precipitation and hydrothermal process yielded core-shell-structured LaSTO nanocubes with Nb-doped surfaces. A violent exothermic reaction facilitated the growth of rapidly formed LaSTO nanocube cores enclosed by (100) facets at room temperature. The Nb-doped surface layers (shells) formed on the core nanocubes during the hydrothermal process. The core-shell nanocubes grew preferentially along the <111> direction, and they have different shapes with increasing Nb doping levels, changing from cubic shapes with sharp edges to concave cubic shapes. The core-shell-structured nanocubes assembled in a face-to-face arrangement on SiO_2_/Si substrates by a slow evaporation process. The resultant thin film showed a 10 μm thickness with a flat and smooth surface. This work provides a convenient and practical synthesis process for metal ion doping on the nanoparticle surface, forming core-shell structures in self-assembling nanostructures, enhancing thermoelectric performance due to an energy filtering effect for thermoelectric applications.
